# The rubber foot illusion

**DOI:** 10.1186/s12984-015-0069-6

**Published:** 2015-09-04

**Authors:** Simona Crea, Marco D’Alonzo, Nicola Vitiello, Christian Cipriani

**Affiliations:** The BioRobotics Institute, Scuola Superiore Sant’Anna, viale Rinaldo Piaggio, Pontedera (PI), Italy; Don Carlo Gnocchi Foundation, via di Scandicci, Firenze (FI), 269 Italy

**Keywords:** Rubber foot illusion, Augmenting sensory feedback, Lower-limb prostheses, Amputees

## Abstract

**Background:**

Lower-limb amputation causes the individual a huge functional impairment due to the lack of adequate sensory perception from the missing limb. The development of an augmenting sensory feedback device able to restore some of the missing information from the amputated limb may improve embodiment, control and acceptability of the prosthesis.

**Findings:**

In this work we transferred the Rubber Hand Illusion paradigm to the lower limb. We investigated the possibility of promoting body ownership of a fake foot, in a series of experiments fashioned after the RHI using matched or mismatched (vibrotactile) stimulation. The results, collected from 19 healthy subjects, demonstrated that it is possible to elicit the perception of possessing a rubber foot when modality-matched stimulations are provided synchronously on the biological foot and to the corresponding rubber foot areas. Results also proved that it is possible to enhance the illusion even with modality-mismatched stimulation, even though illusion was lower than in case of modality-matched stimulation.

**Conclusions:**

We demonstrated the possibility of promoting a Rubber Foot Illusion with both matched and mismatched stimulation.

## Introduction

The Rubber Hand Illusion (RHI) is a perceptual illusion causing the feeling of ownership of a realistic rubber hand when placed in full view and synchronously stimulated with the person’s own hand, which is hidden from view [1]. The illusion happens as a result of the interaction and coherence of vision, touch and proprioception [2, 3] and it typically induces a shift of the perceived location of the participant’s hand towards the rubber hand (proprioceptive drift) and a strong skin conductance response (SCR) to a threat stimulus on the rubber hand. This illusion does not occur when the stimulations are not synchronous [1–3].

In recent years the paradigm of the RHI was investigated on different body parts, e.g. the tongue [4], the arm [5] or the entire body [6] and translated from basic neuroscience studies to different application scenarios, like virtual reality [6] or upper-limb prostheses [7–10]. Indeed Ehrsson and colleagues demonstrated that the RHI can be elicited even when the natural hand is missing, namely in upper-limb amputees when stimulated on *referred phantom fingers* on the stump [7]. This finding suggested engineers to develop hand prostheses with tactile sensory feedback capable of promoting the embodiment of the prosthesis itself [7, 8]. In our previous studies we suggested and proofed the possibility of inducing the RHI using mismatched stimulation (i.e. sensory substitution) on healthy subjects [9] and transradial amputees [10]. In particular we used miniaturized vibrotactile stimulators, that are small enough to be embedded in a prosthetic socket, conversely to current state-of-the-art haptic stimulators, too bulky [9].

Lower-limb amputation, similarly to upper-limb loss, causes the individual a huge functional impairment due to the lack of adequate sensory perception from the missing limb. While ambulation is generally a well-learned activity, which requires little or no cognitive effort by healthy adults [11], ambulating with an above-knee prosthesis is a cognitively demanding task, because the normal proprioceptive afferent flow is lost, and amputees find it difficult to control their prostheses, especially in unstructured environment, i.e. uneven terrain. The use of vision combined to a sensory feedback stimulation provided in a natural and intuitive fashion, (e.g. by exploiting the phantom limb sensation, as suggested by Murray [12] and Ehrsson and collegues [7]) could improve the controllability of the prosthesis, as tactile information from the prosthesis would match and enrich the information from the vision. At the same time, an intuitive proprioceptive feedback could also be an important asset in itself; in fact if the prosthesis could induce a feeling of ownership it is predictable that the ratio of unsatisfied patients will reduce [12].

In this work we transferred the RHI paradigm to the lower limb. The first goal of the study, similarly to the work recently published by Lenggenhager and collegues [13], was to investigate the possibility of promoting body ownership of a fake foot, in series of experiment fashioned after the RHI, using matched stimulation. The second goal of the study was to investigate the possibility to enhance the feeling of possessing a rubber foot when applying mismatched (vibrotactile) stimulation, which can be potentially integrated in a sensory feedback system for lower-limb prosthesis. The results, collected from 19 healthy subjects, demonstrated indeed the possibility of promoting a *Rubber Foot Illusion* (RFI) with both matched and mismatched stimulation.

## Materials and methods

**Fig. 1:**
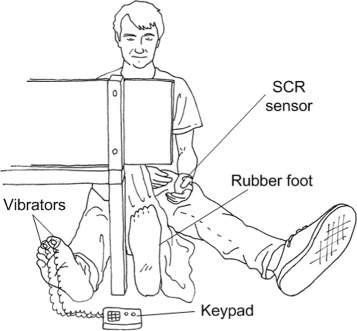
Experimental setup. The participant is looking at the rubber foot, placed in front of him while his real foot is hidden from view

Nineteen able-bodied volunteers (10 females) participated in this study after signing a written informed consent. All experimental procedures were performed according to the standards set by the declaration of Helsinki for medical research involving human subjects. Each participant sat comfortably on a bed with both legs lying in a supine position and with his/her right leg out of sight, behind a screen. The participant was instructed to fix his/her sight on a rubber foot (single-axis foot, Ottobock Gmbh) during the experiment. Modality-matched and modality-mismatched stimulation conditions were tested. During the modality-matched conditions, congruent stimulation was delivered on the real and the rubber feet, using two paintbrushes, as in the original RHI experiment. In the modality-mismatched conditions the stimulation was incongruent; the rubber foot, in full view, was manually stimulated with the paintbrush while the hidden real foot was stimulated by small vibrators taped on the first and second toes, triggered off by a keypad, as in our previous studies [9, 10] (as depicted in (Fig. 1)). Each vibrator (8 mm diameter, 3.4 mm height, 0.7 g weight) could be selectively activated to vibrate at a pre-defined vibration frequency (165 Hz) and force amplitude (0.36 N; i.e. largely supra-threshold) or deactivated. As soon as a specific key on the keypad was pressed, the corresponding unit would start vibrating, until the key was released. The time delay between the pressure of a key and starting of perceivable vibration can be considered negligible (i.e. <10 ms). Paintbrush stimulations on the rubber foot were applied on the areas corresponding to where the vibrators were placed on the real foot, i.e. on the first and second toes. Details on this set-up can be found in [14]. Both the congruent and incongruent conditions were tested with either synchronous or asynchronous stimulation (a temporal delay of about 0.5 s was included between stimulations) for a total of four combinations. The asynchronous conditions were included as control conditions for the synchronous ones. The four combinations of modality and timing (congruent synchronous - CS, congruent asynchronous - CA, incongruent synchronous - IS and incongruent asynchronous - IA) were tested twice for each subject; 50 % of the trials (balanced across conditions) ended with a threat stimulus, i.e. a needle was used to stab the rubber foot, in order to measure the SCR (as in the conventional RHI experiment [7]). Each trial lasted 150 s; in previous studies the stimulation duration was ranged from a minimum of 45 s [9] to a maximum of 240 s [15]. The order of execution of the trials was randomized across the participants.

We quantified the embodiment of the rubber foot by means of the three state-of-art control measures, i.e. self-assessment questionnaires, proprioceptive drift and SCR [15]. The questionnaire (Table 1) comprised of nine statements adapted from [1] for application on the foot instead of on the hand and translated into Italian. Three of the statements, namely illusion statements, referred to the extent of sensory transfer into the rubber foot and self-attribution of it during the trial (S1–S3). The remaining six statements, namely control statements, served as controls, for assessing suggestibility and “placebo effect”. Moreover, in addition to the nine statements, subjects were asked to rate vividness and prevalence of self-attribution of the rubber foot. The vividness was defined as how life-like and realistic the illusion was when it was experienced; it was rated from 0 to 10. The prevalence rating (from 0–100 %) reflected the percentage of time that the illusion was experienced (equivalent to the continuance of the illusion). To assess the proprioceptive drift, the participant had to indicate the position of his/her big toe on a ruler placed over his/her foot, before and immediately after the stimulation. The subtraction of the two measurements resulted in the proprioceptive drift. In order to avoid any memory effect the ruler was positioned with different offsets all the times [15]. When the trial ended with a threat stimulus (i.e. a needle attached to a syringe, which was used to stab the rubber foot), we measured the SCR first, then the proprioceptive drift; in the other trials first we measured the proprioceptive drift then we asked to fill-in the questionnaire.

**Table 1:** Questionnaire. Statements from 1 to 3 are illusion statements. Statements from 4 to 9 are suggestion statements. Statements 10 and 11 are respectively used to quantify the vividness and prevalence of the illusion

	Question	Score
S1	It seemed like I was feeling the stimulation in the point where the rubber foot was being touched	[ −3+3]
S2	It seemed like the stimulation I was feeling was caused by the touch of the paintbrush on the rubber foot	[ −3+3]
S3	I felt like the rubber foot was mine	[ −3+3]
S4	I felt like my (real) foot was moving towards left (towards the rubber foot)	[ −3+3]
S5	It seemed like I had more than one right foot and leg	[ −3+3]
S6	It seemed like the stimulation I was feeling became from some places between my foot and the rubber foot	[ −3+3]
S7	I felt like my (real) foot like was becoming rubbery	[ −3+3]
S8	It seemed like the rubber foot was moving towards right (towards my real foot)	[ −3+3]
S9	The rubber foot started to look like my (real) foot, in terms of shape, skin tone, o other characteristics	[ −3+3]
S10	Using a score from 0 to 10, quantify the vividness (how much your illusion of a rubber foot was realistic)	[0 10]
S11	Using a score from 0 to 100 %, quantify how long you had the illusion that the rubber foot was yours	[0 100]

The Kolgomorov-Smirnov test was used to verify whether the measurements were normally distributed. Then, two-way repeated-measures ANOVA (factors: timing and modality) was performed singularly for each measurement (i.e. questionnaires, drifts or SCR) in order to assess significant differences in the stimulation conditions. Similarly the results were pairwise compared using two-tailed paired t-test [15, 16], with Tukey-Kramer correction. Statistical significance was set to 0.05.

## Results

**Fig. 2:**
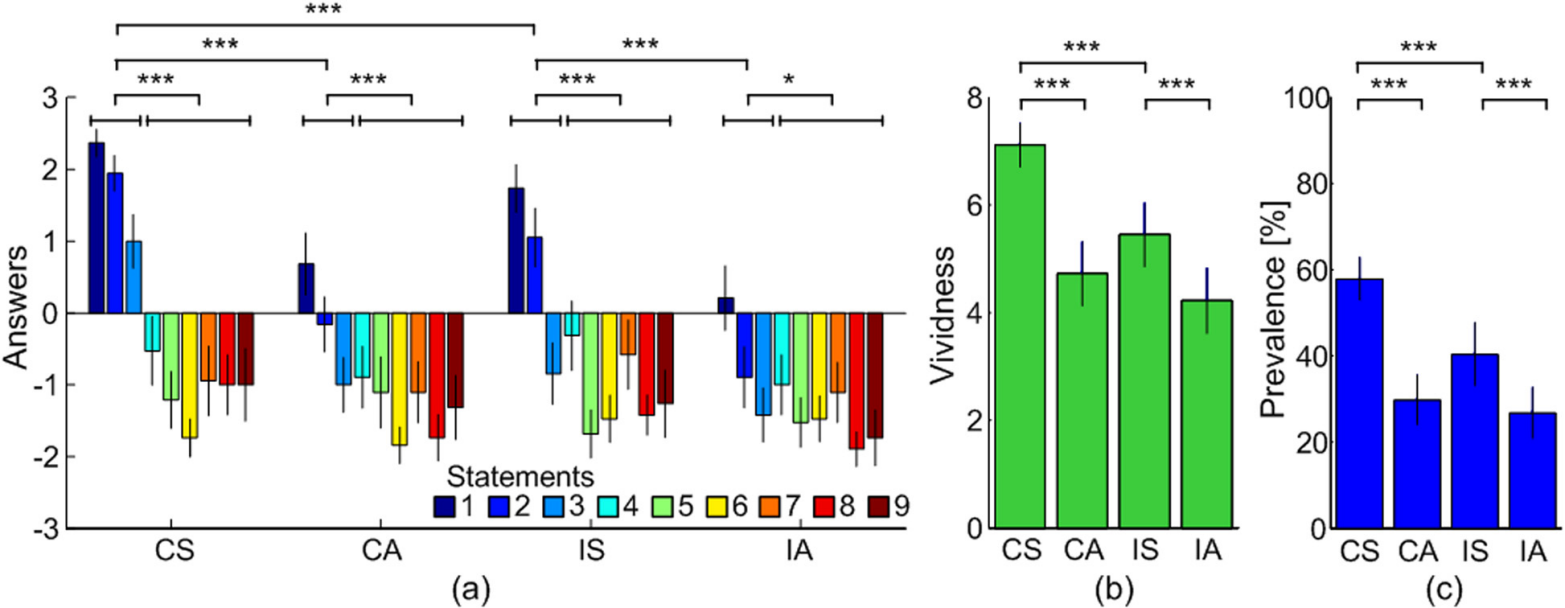
Average ratings of the self-assessment questionnaire. **a** 9 statements. Illusion statements are from 1 to 3. Suggestion statements are from 4 to 9. **b** Vividness of the illusion. **c** Prevalence of the illusion. CS = Congruent Synchronous, CA = Congruent Asynchronous, IS = Incongruent Synchronous, IA = Incongruent Asynchronous. * indicates *p*<.05, *** indicates *p*<.001

Results of the self-assessment questionnaire substantially agree with previous studies on the hand, with illusion statements rated significantly higher than control statements (aimed to assess suggestibility of the subjects) (Fig.2(a)). Statistical differences between the average scores on illusion and suggestion statements (*p*<0.05) were found in all the four experimental conditions (t test, CS, *p*<.0001; CA, *p*=.0003; IS, *p*<.0001; IA, *p*=.0194). Moreover, a two-way repeated-measures ANOVA on average values of illusion statements revealed a significant main effect of timing (*F*(1,18)=76.74,*p*<.0001) and modality (*F*(1,18)=19.81,*p*=.0003). No statistically significant interaction between the effects of timing and modality was found (*F*(1,18)=2.39,*p*=.1395). From the post-hoc multiple comparison with Tukey-Kramer adjustment, both matched and mismatched modalities revealed higher illusion in the synchronous conditions than in asynchronous ones (CS vs CA *p*<.0001, IS vs IA *p*=.0001). Also, in the illusion statements, the modality (congruent vs incongruent) revealed a statistical difference between synchronous conditions, with higher illusion in the congruent than in the incongruent conditions (CS vs IS *p*=.0005; CA vs IA *p*=.0548).

Results from the two-way repeated measures ANOVA showed a statistically significant interaction effect between the timing and modality on the vividness of the illusion (*F*(1,17)=7.37,*p*=.0147) (Fig.2(b)). Significant main effects of timing (*F*(1,17)=70.62,*p*<.0001) and modality were found (*F*(1,17)=25.42,*p*=.0001). Post-hoc pairwise analysis revealed that, in both congruent and incongruent conditions, vividness scored significantly higher when stimulations were synchronous than in case they were asynchronous (t-test with Tukey-Kramer adjustment, CS vs CA *p*<.0001, IS vs IA *p*=.0009). Significantly higher vividness was also found in the CS compared to the IS (t-test with Tukey-Kramer adjustment, *p*<.0001) while no significant difference was found between CA and IA cases (t-test, *p*=.1182).

Two-way repeated-measures ANOVA applied on the prevalence scores (Fig.2(c)) revealed again a significant interaction effect between timing and modality factors (*F*(1,17)=11.02,*p*=.0041), and two main effects for the timing (*F*(1,17)=91.68,*p*<.0001) and the modality (*F*(1,17)=22.31,*p*=.0002). Post-hoc analysis found that prevalence scored higher in synchronous conditions than in asynchronous ones (CS vs CA *p*<.0001; IS vs IA *p*=.0004). The effect of modality was also significant for prevalence scores (*F*(1,17)=22.31,*p*=.0002), and pairwise comparison resulted in higher prevalence in synchronous congruent condition than in synchronous incongruent one (CS vs IS *p*<.0001; CA vs IA *p*=.3346).^1^

**Fig. 3:**
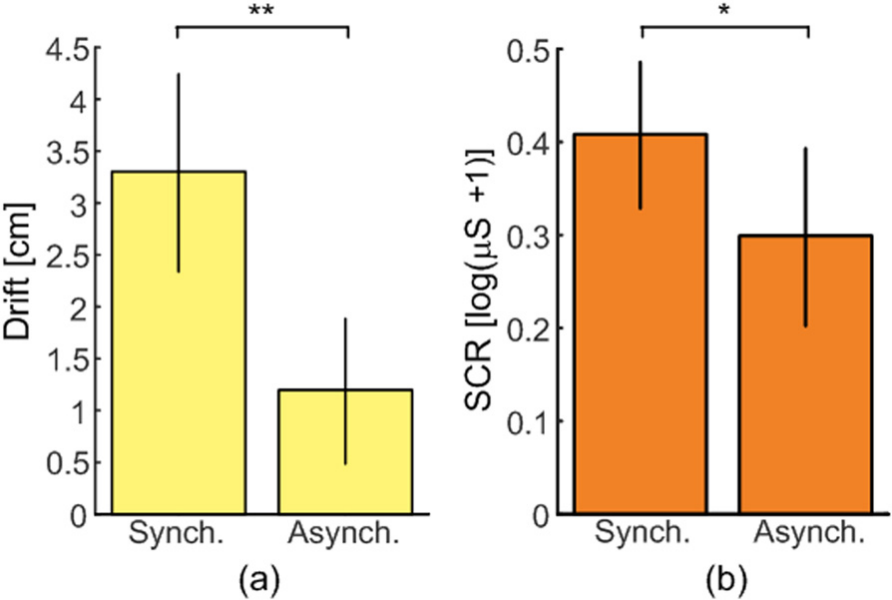
Results of the objective measures of the embodiment. Mean values ± Standard Error of Mean across subjects. **a** Proprioceptive drift. **b** Skin conductance response (SCR). Synch. = Average of synchronous congruent and incongruent results. Asynch. = Average of asynchronous congruent and incongruent results. * indicates *p*<.05, ** indicates *p*<.01

Two-way repeated-measures ANOVA showed no significant interaction effect between timing and modality factors (*F*(1,18)=.33,*p*=.5735) and a significant main effect of timing in the proprioceptive drift results (*F*(1,18)=12.45,*p*=.0024) (Fig.3(a)). Proprioceptive drift in congruent synchronous trial was higher than in the corresponding asynchronous trial (t-test, CS vs CA *p*=.0095), while no difference was found for incongruent conditions (IS vs IA *p*=.0511). In this case, the effect of stimulation modality was not significant and pairwise comparison was not performed.

Regarding the SCR measurements, the two-way ANOVA showed a significant main effect for the timing of the stimulations (*F*(1,18)=6.75,*p*=.0181), no significant main effect of the stimulation modality (*F*(1,18)=4.02,*p*=.0603) and no significant interaction effect (*F*(1,18)=3.5,*p*=.0777) (Fig.3(b)). Pairwise comparison of different timing conditions revealed higher SCR in CS condition than in CA (*p*=.0054), while no difference was found between IS and IA (*p*=.6132). Since modality did not result in a main effect, pairwise comparison was not assessed.

It is worth to recall that combined measurements of embodiment, i.e. questionnaire, proprioceptive drift and the SCR, scored in line with previous studies on the RHI [2, 9, 17].

## Discussion and conclusions

The body schema is usually defined as a continuously updated sensorimotor map of the body that informs the brain about what parts belongs to the body and where those parts are located [18]. The trick of the RHI is a direct consequence of multisensory integration, which basically misleads the brain as to the status of ownership of a fake hand, provoking its inclusion in the body schema. The largest part of neurophysiological studies has explored the body schema using stimulation to and near the hands. Hands may be considered special in that they are used for almost any kind of action we perform, but it seems that processing principles revealed for the hands may not generalize to other body parts [19]. Indeed, combining multisensory information has different implications in case the stimuli come from different parts of the body’s surface. For example tactile information takes different amounts of time to be processed by the brain depending on the distance between the stimulated surface and the brain and some studies showed how the integration of visual and tactile stimulation of the foot can lead to bad perception of simultaneity of the stimuli [20]. Our results provide evidence that it is possible to elicit the perception of possessing a rubber foot when modality-matched stimulations are provided synchronously on the biological foot and to the equivalent rubber foot areas. Indeed, all the three independent measurements of embodiment, classically employed in similar experiments on the RHI [9, 15, 21], gave closely matched results supporting this main outcome, thus confirming that even though visual and tactile stimulations applied on the foot undergo different processing mechanisms, the multisensory integration can still effectively compound the illusion and the brain integrates the fake foot in the body schema. Second to that, we investigated the RFI approach for application in the development of sensory feedback systems for lower-limb prostheses in order to provide the individual with information from the foot sole [22, 23]. Results proved that it is possible to enhance the illusion even with modality-mismatched stimulations, even though illusion was lower than in case of modality-matched stimulation. Notably asynchronous stimulation, which is used as a standard control test, in both modality congruent and incongruent cases did not induce the illusion, as reported also in previous studies [1, 3, 7–9, 13, 15]. These results open important perspectives in the field of lower-limb prosthetics; indeed, vibrotactile elements can be easily integrated into the socket of lower-limb prostheses in order to stimulate the referred phantom foot areas and induce the RFI on a daily basis. This feature could improve the controllability of the prosthesis [24] and enhance the satisfaction of the amputee in using the prosthesis even more [12].

Future studies will be devoted at investigating whether the proposed mismatched-stimulation paradigm can promote the embodiment of the prosthesis in the body schema of lower-limb amputees.

## Endnote

^1^ Eighteen of nineteen participants replied to vividness and prevalence questions.
